# Vincent’s Angina

**Published:** 1919-01

**Authors:** 


***Vincent's Angina.*** By L. F. BARKER and S. R. MILLER, *Journal A. M. A.*, Sept. 7, 1918.

Taking as a text a case of so-called Vincent's angina, the authors give a rather exhaustive description and discussion of the disease and its symptoms, course and treatment. While it is a comparatively harmless and manageable affection if properly recognized and treated, most cases running a benign course and clearing up in one or two weeks, it may assume a much more virulent form, and a number of cases are now on record occurring in civilian and military practice which have terminated fatally. It is caused by a fusiform bacillus associated with a spirochete somewhat thicker than the *Spirochaeta pallida* and staining more readily. The organism is readily distinguished from the *S. dentium* and *S. refringens*. Like the accompanying bacillus, it is anaerobic. The diagnosis is relatively easy. The essential points are the usual disproportion between the appearance of the lesions and the constitutional symptoms, in this respect being very different from diphtheria. The negative laboratory findings, apart from the spirochete and bacillus in the smears from the throat ulcers, and the clinical course of the disease should prevent errors in diagnosis. It might be confused with syphilis or tonsilitis, but careful examination should exclude these. When the Wassermann reaction is positive a double infection exists. As regards diphtheria, the characteristic bacilli of that disease are invariably absent, and, unlike the diphtheria germ, the *B. fusiformis* is gram-negative. The treatment consists in good oral hygiene and local applications of various kinds. There is abundant evidence that the best drug to apply is arsphenamin, especially in the severer cases. A single intravenous injection has caused rapid healing , or local applications of the same, in glycerin or in solution, two or three times daily, have had equally striking results. General measures should include strict isolation, a high caloric diet in liquid or semi-solid form, sodium cacodylate injections daily, and such symptomatic measures as seem required. All authorities warn against the use of mercury, which seems to increase the incidence of the disease and prolong its cure. The disease has been extraordinarily common in the later years of the war among the allied troops, and the cause of much unnecessary and expensive invalidism. The following well established points should be kept in mind: 1. Vincent's disease is in all likelihood a primary peridental gingivitis, occurring frequently in certain areas and likely to develop anywhere in ill-kept mouths. It is associated with characteristic gum lesions, and capable of spreading to any part of the mouth or throat, and the disease is both infectious and contagious. 2. The most common clinical lesions are ulcerations of the tonsils, to which the name Vincent's angina should be restricted. 3. Wherever located the lesions are caused by the activities of the *B. fusiformis* and an associated spirochete — their specificity is as yet unsettled. It is quite likely that they normally are symbiotic saprophytes, capable under certain conditions of causing pathologic changes. 4. The diagnosis from syphilis and diphtheria is simple. Smears from the lesions usually suffice. 5. Cases of uncomplicated Vincent's disease always give a negative Wassermann. 6. The local application of concentrated solutions of arsphenamin is regarded as the best treatment. 7. Prophylaxis is better than cure. Oral sepsis is inexcusable.

